# The effect of phosphate buffer on improving the performance of autothermal thermophilic aerobic digestion for sewage sludge

**DOI:** 10.1039/c8ra00793d

**Published:** 2018-03-02

**Authors:** Ningben Jin

**Affiliations:** Shanghai Environmental Sanitation Engineering Design Institute Co., Ltd Shanghai 200232 China jinningben@126.com +86 21 54085205 +86 21 54085205

## Abstract

The influence of phosphate buffer on the stabilization of sewage sludge was investigated in autothermal thermophilic aerobic digestion (ATAD). A concentration series of 0.005, 0.01, 0.02 and 0.03 mol phosphate buffer for each liter of sludge was adopted. The phosphate buffer significantly enhanced the performance of the ATAD for sewage sludge. The highest VS removal was achieved by the group with 0.01 mol L^−1^ phosphate buffer, and the stabilization time of the sludge was shortened by 9 days compared with that of the control. The group with the optimal dosage obtained the deepest stabilization level of sludge, which was reflected by the distribution of the particle size, and achieved 6.08% VS removal higher than that of the control in the end. Lower concentrations of carbon, nitrogen and phosphate in the supernatant were also achieved by proper dosing compared with those of the control.

## Abstract 2

Enhancement of autothermal thermophilic aerobic digestion for sewage sludge by the addition of phosphate buffer.

## Graphical abstract

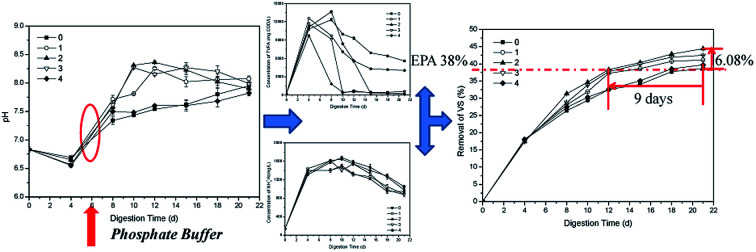

## 1 Introduction

Sewage sludge is produced during the biological treatment process of municipal wastewater, which consists of organic compounds, heavy metals, pathogenic microorganisms and other toxic substances.^1^ Sludge disposed of without treatment would threaten human health through contamination of the environment.^2^ Usually, biological methods are applied in municipal wastewater treatment plants (WWPT) for sludge treatment, including anaerobic digestion and aerobic digestion. Especially, the aerobic digestion is well adopted in the medium- and small-sized WWTPs.^3–5^

Autothermal thermophilic aerobic digestion (ATAD) is an advanced aerobic digestion technology, especially the single stage ATAD with lower cost and simpler control, compared with two-stage ATAD or multiple-stage ATAD.^6^ Usually, the one-stage ATAD is conducted without pH control, and the pH value during the digestion process of sewage sludge always stays between 6 and 9.^7^ In Addition, the pH value in initial phase of one-stage ATAD is always low, due to inefficiency of aeration but high content of organic substances fed for self-heating demand.^8,9^ Especially, accumulation of volatile fatty acid (VFA) would occur when the sludge digestion started with limitation of dissolved oxygen, which would reduce the pH value of the digestion system and then be unfavourable for the digestion process.^10^ Furthermore, the over-produced VFA would also inhibit the dominant microbial population in the one-stage ATAD system.^11^ Overall, the conditioning of pH is essential to improve efficiency of sewage sludge stabilization in the one-stage ATAD process in the initial stage.

The effects of pH on anaerobic digestions have been investigated in many aspects, such as anaerobic digestions with different sources,^12–14^ variant productions,^15–17^ discrepant microbial communities^13,18,19^ and so on. A few research works have also been done about the effect of pH on aerobic digestion, including aspects of nitrification,^20^ biological phosphorus removal,^21^ treatment efficiency, evolution of volatile fatty acids^22^ and so on. Nonetheless, no effort has been made to illustrate the influence of pH on ATAD process.

A solution of sodium hydroxide or chlorine hydride is commonly used as strong alkali or strong acid to adjust the pH during sludge digestion.^15,23,24^ As for buffer solution, phosphate buffer is much more popular than the others.^25,26^ Sodium bicarbonate solution was added as a kind of buffer solution in processes of hydrolysis and VFA production during anaerobic digestion of maize.^27^ The effect of acetate buffer on hydrogen production during fermentation of glucose was also compared with phosphate buffer.^28^ Additionally, sodium sulphate is another good choice for conditioning the pH value.^26^ In summary, the phosphate buffer is adopted to control the pH during the ATAD of sewage sludge in this study, and the effect of phosphate buffer on enhancing performance of one-stage ATAD for sewage sludge is investigated.

## 2 Experimental

### 2.1 Sewage sludge sample

Sewage sludge was collected in a secondary sedimentation tank of a municipal wastewater treatment plant (WWTP) as source for this study. The WWTP located in Shanghai, China, with daily treatment capacity of 45 000 m^3^ wastewater. The sewage sludge sampled would be filtered through a fine sieve with pore size of 0.5 mm. Then the filtrate would be concentrated by centrifugation at 3000*g* for 3 min, in order to obtain feed sludge of total solid (TS) concentration between 5% and 6%. The initial properties of sludge were shown in Table 1.

**Table 1:** Physical–chemical properties of initial sludge employed in simulated one-stage ATAD process^a^

Parameter	pH	TS (g L^−1^)	VS (g L^−1^)	SCOD (mg L^−1^)	TN (mg L^−1^)	NH_4_^+^-N (mg L^−1^)	TP (mg L^−1^)
Value	6.83 ± 0.05	47.9 ± 0.1	33.5 ± 0.1	1017 ± 20	438 ± 17	140 ± 5	265 ± 8

a SCOD – soluble chemical oxidation demand; TN – total nitrogen in supernatant; TP – total phosphate in supernatant; average data and standard deviation obtained from three tests.

### 2.2 Startup of the digestion process

Five cylindrical digesters of tempered glass were utilized to simulate one-stage ATAD systems. The volume of each digester was 5 L, with dimension of 200 mm (*D*) × 400 mm (*H*). The temperature of the digestion process was set to rise from 35 °C to 55 °C at a rate of 5 °C per day, and then maintained at 55 °C afterwards by a circulating water bath heating system.^2^ A flow rate of 0.033 L air/L sludge per hour was supported, with a constant stirring rate of 120 rpm.^29^ A cooling water system was applied in recovery of water vapor in exhaust during the digestion process.

The phosphate buffer of pH 7.5 was prepared due to the pH of one-stage ATAD with good performance was round this pH level.^30^ The buffer was separately added into digesters 6 hours before sampling on 6^th^ day, because both the maximum of VFA and the minimum of pH always happen at this time.^30^ The dosage of phosphate buffer in five digesters were none (group 0), 0.005 mol buffer per L sludge (group 1), 0.01 mol buffer per L sludge (group 2), 0.02 mol buffer per L sludge (group 3), 0.03 mol buffer per L sludge (group 4), respectively, with the reduced amount of sludge by sampling before 6^th^ day counted.

### 2.3 Chemical analysis

The measurements of VS and TS were conducted according to the Standard Method,^31^ with elimination of values result from phosphate addition. The pH was determined by a pH meter (Leici Co. Ltd., Shanghai). Other indicators including soluble chemical oxidation demand (SCOD), total nitrogen (TN), NH_4_^+^-N and total phosphate (TP) were measured with filtrate of sludge samples, as well as volatile fatty acids (VFAs). The filtrate was obtained by filtration of the sludge samples through a mixed cellulose ester membrane (0.45 μm), after centrifugation at 12 000*g* for 5 min. The values of NH_4_^+^-N, SCOD, TN and TP were determined in accord to the Standard Methods.^31^ As for the measurement of VFAs, the filtrate was mixed with 3% H_3_PO_4_ before analysis, to make the pH of the mixed solution stable at approximately 4.0. Then the values of VFAs were measured in a gas chromatograph (Shimadzu GC-2010) in accord to method,^32^ with a flame ionization detector and DB-FFAP column (30 m × 0.25 mm × 0.25 mm). The particle size distributions of sludge on 21^st^ day were analyzed by laser particle size analyzer (BECKMAN COULTER, Delsa Nano C).

All of the indicators were measured in triplicate and the standard deviations were obtained. The software SPSS of version 19.0 for Windows (SPSS, IBM) was applied for statistical analysis, and statistically significant correlations were decided at a confidence interval of 95% (*P* < 0.05; Tukey's test).

## 3 Results and discussion

### 3.1 VS removals with different dosages of phosphate buffer

**Fig. 1:**
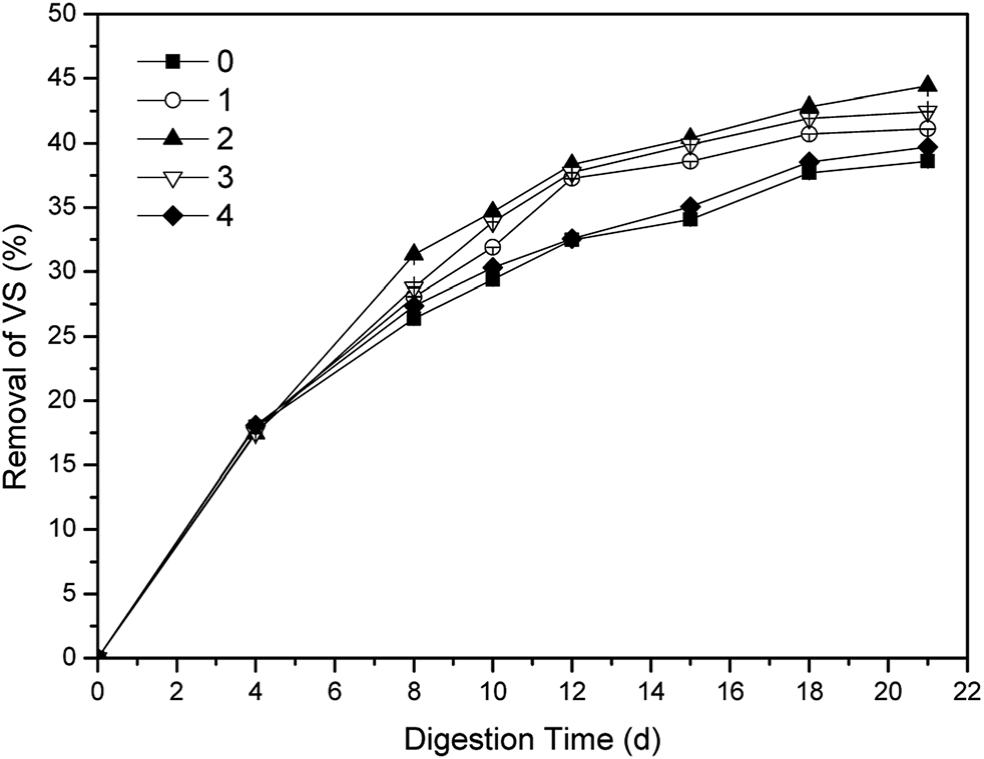
Variations of VS removals with different dosages in one-stage ATAD system.

The performance of aerobic digestion is always evaluated by VS removal, that is why VS removal is important to the assessment of one-stage ATAD.^2^ The effects of phosphate buffer with different dosages on the VS removals were shown in Fig. 1. In the first four days, there were almost no difference of sludge digestion efficiencies among five groups (*p* < 0.05). With the addition of phosphate buffer on 6^th^ day, the VS removals of dosing groups were all higher than that of the control, though the VS removal of group 4 had few advantages over that of the control. After 12 days' digestion, the VS removals of the other three groups were all much higher than that of the control. The group 2 obtained the highest VS removal with 44.42% after 21 days' digestion, while the VS removal of the control was barely 38.58%. Thus, the VS removals of all the groups met the EPA Class A requirements (>38%) for sewage sludge.^33^ The group 2 had finished the stabilization of sewage sludge on 12^nd^ day with 38.34% VS removal, which was 9 days advanced compared with that of the control. The reduction of sludge stabilization time of the group 2 was approximate to the result of ammonia disinhibition^7^ and VFA disinhibition,^10^ which should owe to the relief of inhibition by over-produced ammonia and VFA. In addition, neither insufficient nor excessive dosage of the buffer was unfavorable for the digestion of sewage sludge. In a word, the group 2 with optimal dosage had the best effect on stabilization for sewage sludge.

### 3.2 Variations of pH values, TVFA and NH_4_^+^-N with different dosages of phosphate buffer

**Fig. 2:**
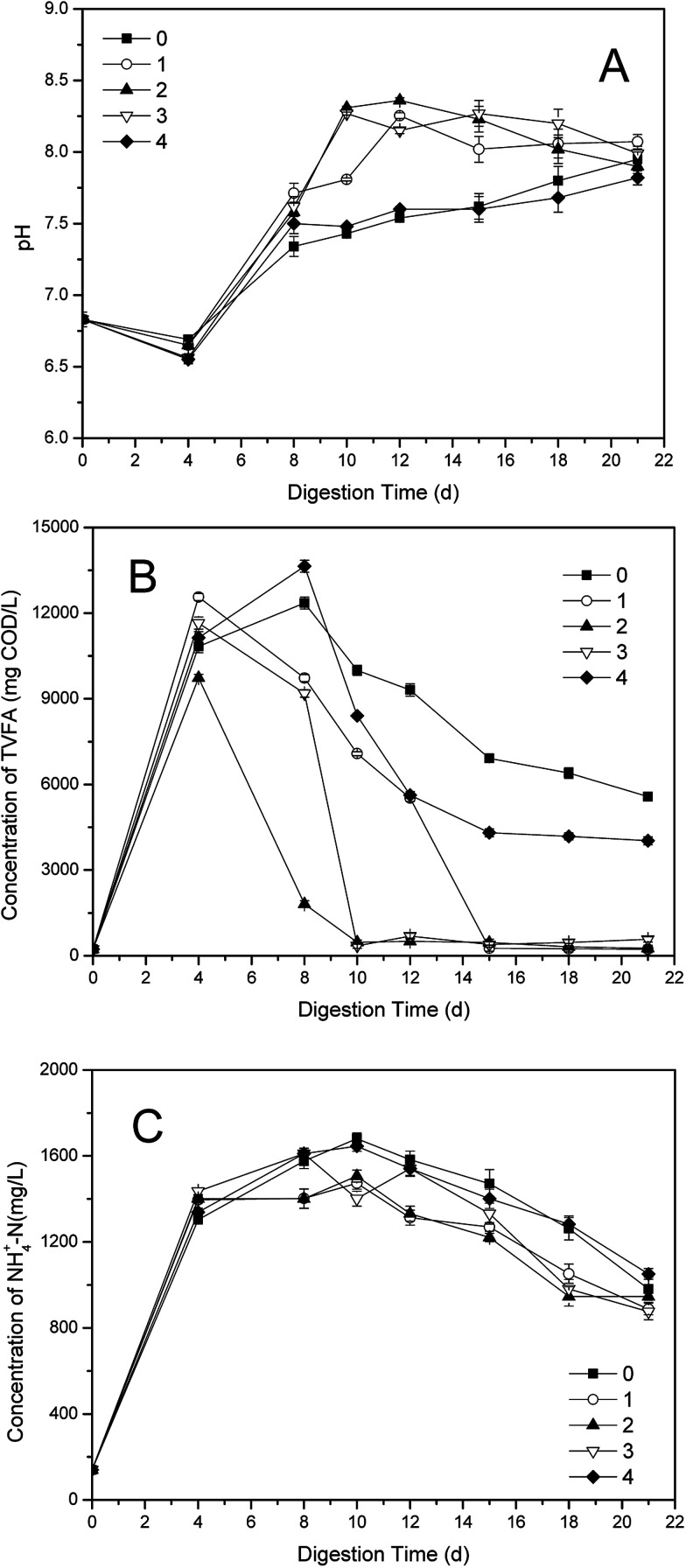
Variations of (A) pH; (B) TVFA and (C) NH_4_^+^-N concentrations in supernatant with different dosages in one-stage ATAD system.

The effects of different dosages of phosphate buffer on the pH were illustrated in the Fig. 2A. In the initial stage of single-stage ATAD (0–4 d), the pH values in all groups decreased due to the large amount of VFA produced during hydrolysis phase.^2^ Then the pH increased along with the consumption of VFA and accumulation of NH_4_^+^-N,^10^ such as the tendency of pH value in the control. However, the groups with the buffer all had rapid increases after reagents additions, especially the pH value in the group 2, except for group 4. As shown in Fig. 2A, after a decline at the beginning, both of the pH values in the control and the group 4 increased until the end of digestion. But the pH values in the other three groups declined after 12^nd^ day, though the decreases of pH values were within a small range. The highest value of pH was 8.36, obtained in the group 2 on 12^nd^ day, within the pH scope of phosphate buffer. After 21 days' digestion, the disparity of pH values in five groups reduced and fluctuated in a range of 7.8–8.1, according with the result reported.^8^ The pH value in the control was higher than that of the group 4 after 15^th^ day, which should be due to the overdosage of phosphate buffer.

Generally, the pH value in one-stage ATAD is influenced by the balance between VFA and NH_4_^+^-N.^30^ Total VFA (TVFA) consist of acetic acid, propionic acid, *n*-butyric acid, iso-butyric acid, *n*-valeric acid and iso-valeric acid constituted.^8^ As shown in Fig. 2B, the TVFA concentration in the control reached maximum on 8^th^ day, with 12 355 mg COD per L. However, the TVFA contents in dosing groups all declined with the additions of the buffer, except for the group 4. Particularly, the TVFA values in the group 2 had a sharp decrease after 6^th^ day, and the TVFA value was very low after 10 days' digestion, beneath 500 mg COD per L. It was consistent with the rapid rate of VS removal in the group 2, for that VFA are degradable intermediate products during the digestion of sewage sludge,^30^ which were similar to those happened in the group 1 and the group 3. In the late stage of digestion, the TVFA values of the control and the group 4 were fluctuated, with difference value of 1000–2000 mg COD per L.

The variations of NH_4_^+^-N concentrations with different dosages of phosphate buffer were illustrated in Fig. 2C. In the first four days, the NH_4_^+^-N contents in all digesters increased very quickly, because of the decomposition and release of protein in roughly changed environment when the temperature of the system began to warming up.^34^ Then the NH_4_^+^-N value of the control increased highest of all until the end of digestion. The rising rates of NH_4_^+^-N concentration in the dosing groups slowed down when the buffer added, especially that of the group 2 which maintaining the least throughout the late stage. The different value between the control and the dosing groups was 100–200 mg L^−1^ throughout the whole digestion process.

The VFAs could be reduced by degradation, however, the NH_4_^+^-N would be held back by the phosphate buffer, but decrease of NH_4_^+^-N by neither utilization nor volatilization was hampered. Therefore, higher consumption rate of TVFA but lower reduction rate of NH_4_^+^-N would contribute to the faster rise of pH value after addition of phosphate buffer, compared with that of the control. Nevertheless, the block of NH_4_^+^-N did not make the pH level of the one-stage ATAD system much high, owing to the buffer action of phosphate solution.

### 3.3 Effect of phosphate buffer on carbon and nitrogen as well as phosphate

**Fig. 3:**
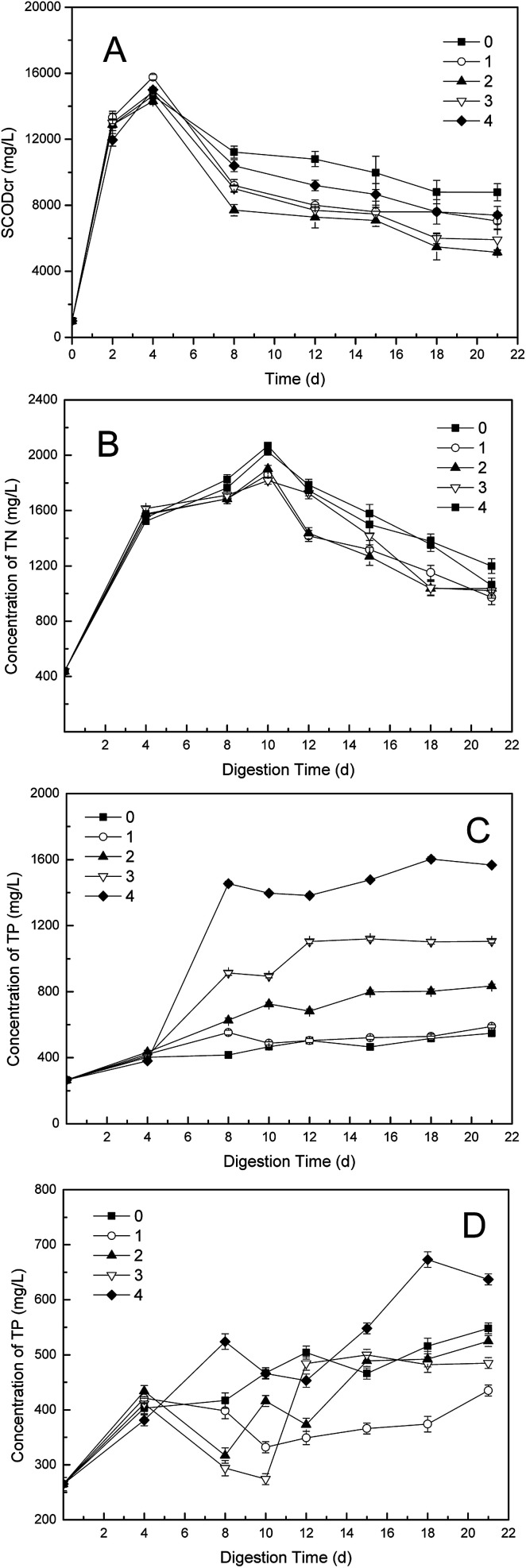
Variations of (A) SCOD; (B) TN; (C) TP + PB and (D) TP − PB concentrations in supernatant with different dosages in one-stage ATAD system. Phosphate buffer was defined as “PB”.

Variations of SCOD concentrations with different dosages of phosphate buffer in the one-stage ATAD were shown in Fig. 3A. The trend of SCOD was much similar to that of TVFA, for the TVFA is commonly main component of the SCOD.^30^ All groups had shared the same tendency, and the group 2 obtained the lowest value after addition of the buffer. Although the SCOD in the control began to decrease on 8^th^ day, the SCOD in other dosing groups reduced much more quickly, which should be ascribed to the promotion of biodegradation by phosphate buffer. Less than 4000 mg COD per L was consumed extra, almost 50% reduction achieved, comparing the SCOD value in the control with that in the group 2. This find was well coincident with the change of TVFA.

The changes of TN contents with different dosages of phosphate buffer were illustrated in Fig. 3B. The TN values in all digesters showed no obvious differences in the first four days (*p* < 0.05). Then the divergences emerged when the buffer added on 6^th^ day, and the increase rate of TN values in the dosing groups slowed down compared with that of the control. After 10 days' digestion, the TN values in all groups started to decrease, likes the situation happened to the NH_4_^+^-N values, for that the TN mainly consisted of NH_4_^+^-N.^30^ Difference value of 100–300 mg L^−1^ TN contents between the control and the dosing groups was found, which was in coincidence with that of NH_4_^+^-N.

The effects of phosphate buffer on the phosphate values during one-stage ATAD process were shown in Fig. 3C. There were almost no differences of TP contents among five digesters in the first four days (*p* < 0.05). Then the TP values were enlarged with additions of phosphate buffer, and higher concentration of TP was obtained as more content of the buffer added obviously. Thus the input of phosphate by addition of phosphate buffer could not be eliminated completely through metabolism of microbes. Nonetheless, as seen in Fig. 3D, the TP level in each dosing group, was much lower than that of the control, except for that of the group 4, if the phosphate brought in by addition was not included. This find was in accord with the result of VS removal, because that the one-stage ATAD was a closed system for phosphate during digestion of sewage sludge, which meant that the reduction of phosphate could only be utilization to synthesis of adenosine triphosphate (ATP) by microbes.^30^ Although the TP content in the group 1 was lower than that in the group 2, it should be due to the slower release of TP from disintegration of microorganisms. This result could also be found out by comparison of TP values in the control and the group 1. Hence, in view of the VS removal, the addition of phosphate buffer neither hindered the release nor utilization of TP by microbes, on the contrary, it promoted these processes during the digestion of sewage sludge.

### 3.4 Particle size distributions of final digestion sludge with different dosages of phosphate buffer

**Fig. 4:**
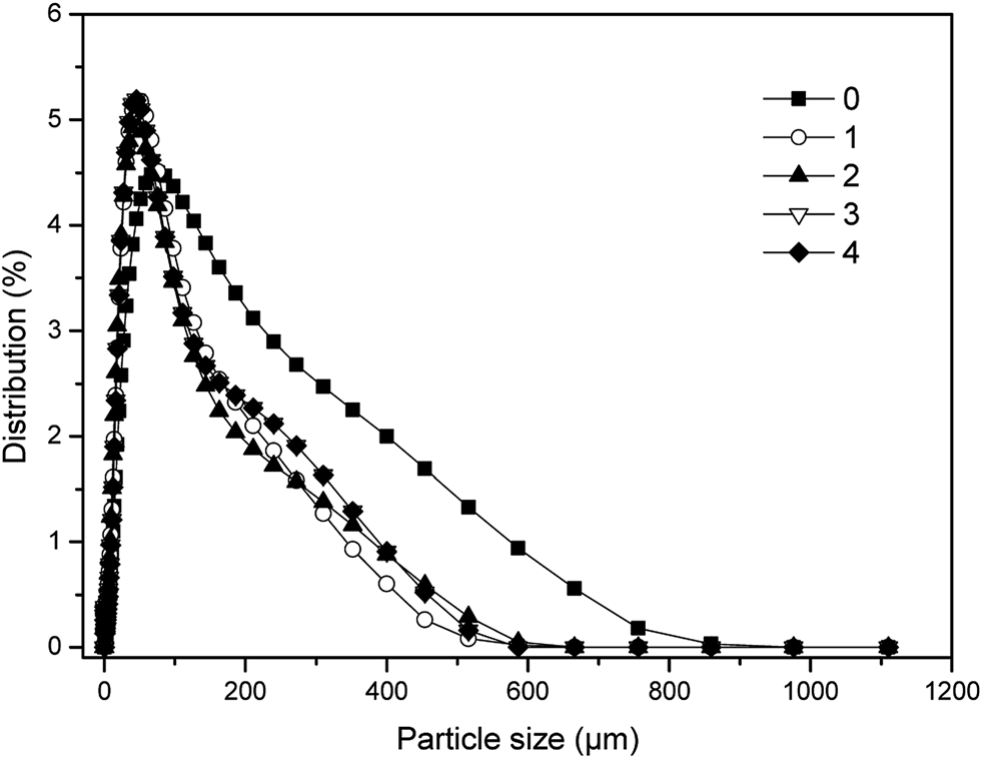
Particle size distributions of final digestion sludge with different dosages in one-stage ATAD system.

The distributions of particle size of sludge at the end of digestion in five digesters were illustrated in Fig. 4. Obviously, all of the five samples shared the same particle size (186 μm) with the largest proportion, respectively. However, the percentage of the sludge with the particle size of 186 μm in the group 2 was least among all digesters (2.04%). While the percentage of the sludge with the particle size of 186 μm in the control was highest of all (3.36%). In addition, the distributions of particle size of sludge in dosing groups were much more concentrated, and closer to the smaller size than that of the control. Especially, the particle size of sludge in the group 2 was the smallest, and its corresponding proportion was least, which should be contributed to the fastest and deepest level of stabilization for sewage sludge. Furthermore, the optimal dosage of phosphate buffer should be helpful to the best distribution of particle size, for that maintaining the most suitable electronegativity of sludge particles, which was best for the mass transfer as well as metabolism of microbes.^35^

## 4 Conclusion

Utilization of phosphate buffer had been proved to be a feasible and effective approach to enhance stabilization of sewage sludge in one-stage ATAD. The optimal dosage of phosphate buffer was 0.01 mol buffer per L sludge, and the group 2 had the highest VS removal with 44.42% in the end, which was 6.08% higher than that of the control. The group 2 obtained 9 days ahead of stabilization time compared with that of the control, and achieved the deepest level of stabilization which reflected on distributions of particle size of the final digestion sludge as well. Lower contents of carbon, nitrogen and phosphate in the supernatant were also found in the group 2.

## Conflicts of interest

There are no conflicts to declare.

## Supplementary Material

## References

[cit1] Liu Y. J., Gao M., Zhang A. N., Liu Z. (2017). Bioresour. Technol..

[cit2] Liu S. G., Zhu N. W., Li L. Y. (2011). Chem. Eng. J..

[cit3] Xia S. Q., Zhou Y., Eustance E., Zhang Z. Q. (2017). Sci. Rep..

[cit4] Zhou Y., Zhang J., Zhang Z. Q., Zhou C., Lai Y. J. S., Xia S. Q. (2017). Chem. Eng. J..

[cit5] Zhang Z. Q., Zhou Y., Zhang J., Xia S. Q., Hermanowicz S. W. (2016). Chem. Eng. J..

[cit6] Layden N. M., Kelly H. G., Mavinic D. S., Bartlett J., Moles R. (2007). J. Environ. Eng. Sci..

[cit7] Yuan H. P., Xu C. W., Zhu N. W. (2014). Bioresour. Technol..

[cit8] Liu S. G., Song F. Y., Zhu N. W., Yuan H. P., Cheng J. H. (2010). Bioresour. Technol..

[cit9] Cheng J. H., Zhu J., Kong F., Zhang C. Y. (2015). J. Environ. Manage..

[cit10] Jin N. B., Jin B., Zhu N. W., Yuan H. P., Ruan J. B. (2015). Bioresour. Technol..

[cit11] Jin N. B., Shou Z. Q., Yuan H. P., Lou Z. Y., Zhu N. W. (2016). Bioresour. Technol..

[cit12] Jiang J. G., Zhang Y. J., Li K. M., Wang Q., Gong C. X., Li M. L. (2013). Bioresour. Technol..

[cit13] Jie W. G., Peng Y. Z., Ren N. Q., Li B. K. (2014). Bioresour. Technol..

[cit14] Jankowska E., Chwialkowska J., Stodolny M., Oleskowicz-Popiel P. (2015). Bioresour. Technol..

[cit15] Zhai N. N., Zhang T., Yin D. X., Yang G. H., Wang X. J., Ren G. X., Feng Y. Z. (2015). Waste Manag..

[cit16] Dareioti M. A., Vavouraki A. I., Kornaros M. (2014). Bioresour. Technol..

[cit17] Wang K., Yin J., Shen D. S., Li N. (2014). Bioresour. Technol..

[cit18] Huang L., Chen B., Pistolozzi M., Wu Z. Q., Wang J. F. (2014). Bioresour. Technol..

[cit19] Maspolim Y., Zhou Y., Guo C. H., Xiao K. K., Ng W. J. (2015). Bioresour. Technol..

[cit20] Dold P. L. , Ekama G. A. and Marais G. V. R., Instrumentation and Control of Water and Wastewater Treatment and Transport Systems Proceedings of the 4^th^ IAWPRC Workshop Held in Houston and Denver, U.S.A., 1985

[cit21] Nittami T., Oi H., Matsumoto K., Seviour R. J. (2011). New Biotechnol..

[cit22] Ugwuanyi J. O., Harvey L. M., McNeil B. (2005). Bioresour. Technol..

[cit23] Espinoza-Escalante F. M., Pelayo-Ortíz C., Navarro-Corona J., González-García Y., Bories A., Gutiérrez-Pulido H. (2009). Biomass Bioenergy.

[cit24] Chen Y. G., Xiao N. D., Zhao Y. X., Mu H. (2012). Bioresour. Technol..

[cit25] Wang C. H., Lin P. J., Chang J. S. (2006). Process Biochem..

[cit26] Jin Q. R., Jia G. Q., Wang X. L., Li C. (2013). Chin. J. Catal..

[cit27] Cysneiros D., Banks C. J., Heaven S., Karatzas K. A. (2012). Bioresour. Technol..

[cit28] Xu J. F., Mi Y. T., Ren N. Q. (2016). Electron. J. Biotechnol..

[cit29] Xu C. W., Yuan H. P., Lou Z. Y., Zhang G. F., Gong J. Z., Zhu N. W. (2013). Bioresour. Technol..

[cit30] Liu S. G., Zhu N. W., Li L. Y. (2012). Bioresour. Technol..

[cit31] APHA , AWWA and WEF, Standard Methods for the Examination of Water and Wastewater, American Public Health Association/American Water Works Association/Water Environment Federation, Washington, DC, U.S.A., 21st edn, 2005

[cit32] Chen Y. G., Jiang S., Yuan H. Y., Zhou Q., Gu G. W. (2007). Water Res..

[cit33] USEPA , 40 CFR Part 503, Standards for the Use or Disposal of Sewage Sludge, United States Environmental Protection Agency, Washington, DC, 1993

[cit34] Liu S. G., Zhu N. W., Ning P., Li L. Y., Gong X. D. (2012). Chem. Eng. J..

[cit35] Kosman D. J. (2013). Coord. Chem. Rev..

